# Editorial: Understanding and targeting neuro-immune interactions within disease and inflammation

**DOI:** 10.3389/fimmu.2023.1201669

**Published:** 2023-04-18

**Authors:** Eric H. Chang, Daniela Carnevale, Sangeeta S. Chavan

**Affiliations:** ^1^ Laboratory for Biomedical Sciences, Institute of Biolectronic Medicine, Feinstein Institutes for Medical Research, Northwell Health, Manhasset, NY, United States; ^2^ Donald and Barbara Zucker School of Medicine at Hofstra/Northwell, Hempstead, NY, United States; ^3^ Elmezzi Graduate School of Molecular Medicine, Manhasset, NY, United States; ^4^ Department of Molecular Medicine, Sapienza University of Rome, Rome, Italy; ^5^ Department of Angiocardioneurology and Translational Medicine, IRCCS Neuromed, Pozzilli, Italy

**Keywords:** vagus nerve, cytokine signaling, checkpoint inhibition, multiple sclerosis and neuroimmunology, microglia, aquaporin2 (AQP2), carotid body (CB), pancreatitis

## Introduction

Research at the intersection of the nervous system and immune system is growing rapidly and holds significant potential for understanding and treating various diseases and inflammatory conditions. Although these two systems were traditionally studied independently, a body of growing evidence has shown that they are intricately linked and that changes in one system can significantly impact the other. Particularly in the context of disease and inflammation, interactions between the nervous and immune systems play a crucial role in the initiation, progression, and resolution of pathological states, including autoimmune diseases, neuroinflammatory conditions, and neurodegenerative disorders.

The study of neuro-immune interactions and neuroimmunology originally emerged from clinical observations dating back to the 17^th^ Century on patients with neuroinflammatory conditions that fit the description of what we now call multiple sclerosis (1). These early observations indicated that immune responses were present in the CNS and the periphery. As our subsequent understanding of neurology, neuroscience, and immunology improved, multiple lines of evidence emerged that the immune system and nervous system were intimately connected to one another. For example, it was shown that viruses play a role in neurodegenerative diseases, such as Alzheimer’s disease (2), and that immunosuppressants could be used to treat symptoms of multiple sclerosis [3]. Other studies showed that immunosuppression itself could be behaviorally conditioned [4] and that immunoregulatory responses to cytokines changed specific neurons in the brain (5). Collectively, these early findings in the nascent field of neuroimmunology paved the way for the discovery of signaling pathways and an expansive range of neuro-immune interactions that are fundamental to health and disease. There is now ample evidence that the innate and adaptive immune systems interact with a variety of non-immune cells, including neurons, to maintain homeostasis in a range of tissue systems (6, 7, 8). These neuro-immune interactions are incredibly complex and require research approaches from multiple disciplines to fully understand. Here, we have arranged this Research Topic to provide a small snapshot of the broad diversity within this rapidly expanding field.

As the first observations in neuroimmunology were linked to multiple sclerosis, this is a disorder that has remained closely associated with this field of study. And indeed, the Perspective article by Melnikov et al is focused on multiple sclerosis; specifically on the repurposing of serotonergic drugs that modulate immune mechanisms underlying pathogenesis in the disease. Another area of research traditionally associated with neuroimmunology is neuroinflammation involving the resident immune cells of the brain, such as microglia. In this Research Topic, Zhou et al report that the tumor suppressor phosphatase and tensin homolog (PTEN) in microglia plays an important role during cortical development. The deletion of PTEN in microglia leads to impaired synaptic circuits in the developing cortex and behavioral sociability deficits in mice. Manenti et al provide a Review discussing the role of immune checkpoint inhibitors in neuroinflammatory pathways and focus on the programmed cell death protein-1/programmed death-ligand 1 pathway (PD-1/PD-L1), which has been shown to regulate the immune response. Microglia and astrocytes also play potentially important roles in the immune response following brain injury. Deng et al report that aquaporin-2 (AQP2) levels are lower in patients with intracerebral hemorrhage. They also show that overexpression of AQP2, an astrocytic water channel that has been linked to inflammation, induces astrocyte activation and increased secretion of the cytokine interleukin-1 in a rat cell line.

The other articles within this Research Topic reflect a small part of the broad-ranging diversity and interesting new avenues of research in the growing field of neuro-immune interactions. For example, Ahmed et al report that noninvasive ultrasound-based neuromodulation of the spleen attenuates inflammation in a model of pneumonia. By using noninvasive focused ultrasound to stimulate the splenic cholinergic anti-inflammatory pathway (CAP) at different times post-infection, they show that cytokine inhibition can be activated at different levels, and at different times, throughout an immune response to infection. In another study targeting the CAP, Thompson et al use optogenetic stimulation of cholinergic neurons in the brainstem dorsal motor nucleus to reduce inflammation in a model of pancreatitis. They show that selective activation of cholinergic vagus nerve fibers during pancreatitis attenuates tissue damage and reduces inflammatory molecules. Novel tools to manipulate neuro-immune circuits, such as focused ultrasound and optogenetics, will help us gain a better understanding of the specific molecular and cellular components involved in these pathways. Another important, but generally understudied, topic in neuroimmunology is the sensing of inflammatory mediators within the body. Katayama et al review the idea that the carotid body is more than a blood oxygen sensor, but is actually an important multimodal sensing organ that can detect a wide range of circulating molecules, including inflammatory mediators. Together, these articles highlight how important new tools and viewpoints can move a field forward to provide a more granular, and nuanced, understanding of complex neuro-immune interactions.

## Conclusions

The nervous system and immune system are both essential to organism survival. The nature of their rich interactions likely reflects shared evolutionary pressures that drove them to shared signaling systems, such as cytokines and transient receptor potential channels, that have important functions in both systems (9, 10). As our collective understanding of the bidirectional interactions that take place between the nervous system and immune system improves, we will be able to leverage neuroimmunology findings into new therapies to treat both neurological and immunological disorders. The potential to target these interactions for therapeutic purposes has sparked a growing interest in the development of novel strategies for treating a range of conditions, from infections and neurological disorders to chronic inflammation. Despite the extensive progress that has been made, there is still much to be learned about the mechanisms underlying neuro-immune interactions and the optimal strategies for targeting them. We thank the authors for their contributions to this Research Topic and look forward to continued advancements in this rapidly developing field of study.

## Author contributions

EC wrote the first draft of the manuscript. DC and SC edited and provided feedback on the manuscript. All authors approved the final version.
